# Poly[(μ_5_-2-methyl-3,5-dinitro­benzoato)sodium]

**DOI:** 10.1107/S1600536810000498

**Published:** 2010-01-09

**Authors:** Muhammad Danish, Iram Saleem, Nazir Ahmad, Abdul Rauf Raza, Wojciech Starosta, Janusz Leciejewicz

**Affiliations:** a Department of Chemistry, University of Sargodha, Sargodha 40100, Pakistan; b Institute of Nuclear Chemistry and Technology, ul.Dorodna 16, 03-195 Warszawa, Poland

## Abstract

In the crystal of the title coordination polymer, [Na(C_8_H_5_N_2_O_6_)]_*n*_, the Na(I) ion is linked to five nearby anions. Their bonding modes are three monodentate carboxyl­ate O atoms, one *O*,*O*′-bidentate carboxyl­ate group and one *O*,*O*′-bidentate nitro group. This results in an irregular NaO_7_ coordination geometry for the metal ion. This connectivity leads to a layered network propagating in (100).

## Related literature

For the structure of a trimethyl-tin complex with the *ortho*-toluate ligand, see: Danish *et al.* (2010 ▶).
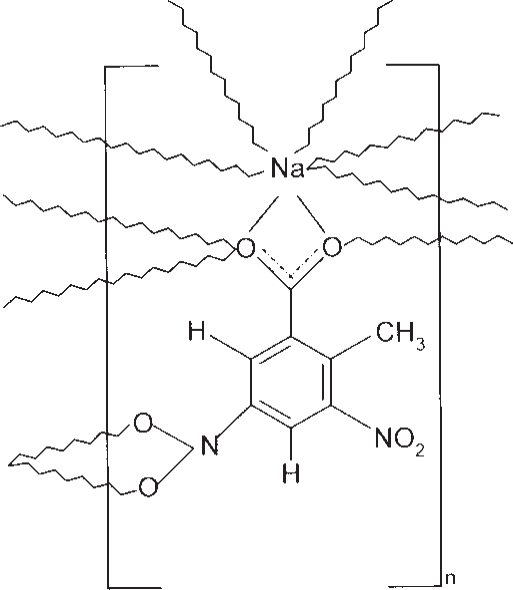


## Experimental

### 

#### Crystal data

[Na(C_8_H_5_N_2_O_6_)]
*M* *_r_* = 248.13
Orthorhombic, 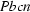

*a* = 27.8428 (13) Å
*b* = 10.452 (2) Å
*c* = 6.642 (6) Å
*V* = 1932.8 (17) Å^3^
*Z* = 8
Mo *K*α radiation
μ = 0.18 mm^−1^
*T* = 293 K
0.42 × 0.14 × 0.08 mm

#### Data collection

Kuma KM-4 four-circle diffractometer
Absorption correction: analytical (*CrysAlis RED*; Oxford Diffraction, 2008 ▶) *T*_min_ = 0.975, *T*_max_ = 0.984
2659 measured reflections
2406 independent reflections
1273 reflections with *I* > 2σ(*I*)
*R*_int_ = 0.047
3 standard reflections every 200 reflections  intensity decay: 0.01%

#### Refinement

*R*[*F* ^2^ > 2σ(*F* ^2^)] = 0.037
*wR*(*F* ^2^) = 0.132
*S* = 1.00
2406 reflections
155 parameters
H-atom parameters constrained
Δρ_max_ = 0.33 e Å^−3^
Δρ_min_ = −0.30 e Å^−3^

### 

Data collection: *KM-4 Software* (Kuma, 1996 ▶); cell refinement: *KM-4 Software*; data reduction: *DATAPROC* (Kuma, 2001 ▶); program(s) used to solve structure: *SHELXS97* (Sheldrick, 2008 ▶); program(s) used to refine structure: *SHELXL97* (Sheldrick, 2008 ▶); molecular graphics: *SHELXTL* (Sheldrick, 2008 ▶); software used to prepare material for publication: *SHELXL97*.

## Supplementary Material

Crystal structure: contains datablocks I, global. DOI: 10.1107/S1600536810000498/hb5280sup1.cif

Structure factors: contains datablocks I. DOI: 10.1107/S1600536810000498/hb5280Isup2.hkl

Additional supplementary materials:  crystallographic information; 3D view; checkCIF report

## Figures and Tables

**Table 1:** Selected bond lengths (Å)

Na1—O1	2.4567 (19)
Na1—O2	2.780 (2)
Na1—O2^i^	2.3571 (17)
Na1—O1^ii^	2.364 (3)
Na1—O2^iii^	2.383 (3)
Na1—O22^iv^	2.6102 (19)
Na1—O21^iv^	2.635 (2)

Symmetry codes: (i) 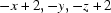; (ii) 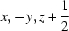; (iii) 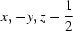; (iv) 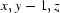.

**Fig. 1.**
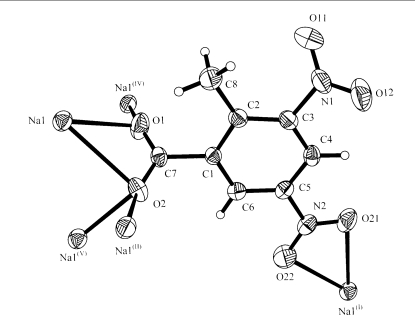
A structural unit of (1) with 50% probability displacement ellipsoids. Symmetry code: (I)x,y + 1,z; (II) -x + 2,-y,-z + 2; (III) -x + 2,y,-z + 3/2; (IV)x,-y,z - 1/2; (V)x,-y,z + 1/2; (VI)x,y - 1,z.

**Fig. 2.**
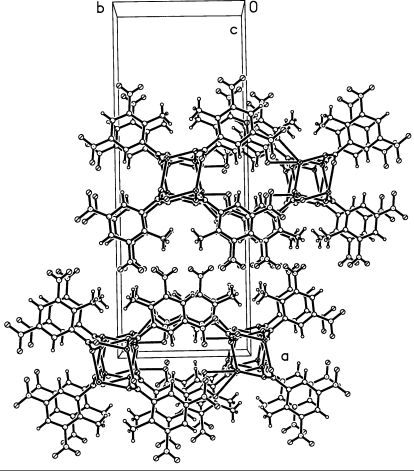
Packing diagram of the structure.

## supplementary crystallographic information

### Comment

The structure of compound (1) is composed of molecular sheets in which Na(I) ions are bridged by ligand carboxylate and nitro-group O atoms. The carboxylate O1 atom acts as bidentate and chelates Na1 and Na1(IV) ions, the O2 atom is bonded to Na1(II) and Na1(V) ions and to the Na1 ion at a longer distance of 2.780 (2) Å.  The O1, O2, Na and Na^(II)^ ions form a distorted plane [r.m.s. 0.0261 (2) Å], the O1 atom chelates the Na^(IV)^ ion below this plane, the O2 atom - the Na^(V)^ion above, giving rise to a molecular column. However, when distances between atoms from a nitro-group of an adjacent ligand to the Na ion are accounted for, the columns form molecular sheets. The coordination geometry of the Na1 ion is represented by a strongly distorted eight-faced polyhedron with an equatorial plane formed by carboxylate O1 and O2(II), nitro O21(VI) and O22(VI) atoms and Na1 [r.m.s. 0.1217 (2)Å]. Carboxylate O2(IV) is at an apex on one side, O2 and O1(V) form two apices on the other side of the equatorial plane. The toluene ring is planar [r.m.s. 0.0070 (2) Å], the carboxylate group makes with it a dihedral angle of 78.0 (2)*^o^*, the nitro-groups - dihedral angles of 42.0 (2)*^o^* (N1/O11/O12) and 9.5 (2)*^o^* (N2/O21/O22). The sheets are held together *via* weak interactions of the van der Waals type since the closest approach between two atoms from adjacent sheets is 3.54 (4) Å.

### Experimental

0.0119 mol of 3,5-dinitro-*ortho* toluic acid was suspended in 15 ml of distilled water contained in a round-bottom flask. Then, 0.0119 mol of an aqueous solution of sodium bicarbonate was added drop-wise with stirring. The mixture was refluxed for 3 h and concentrated to half of its volume, then left at room temperature. Crude crystals appeared within a week. Yellow needles of (I) crystals were obtained by recrystallization from a water/ethanol 3:1 mixture at room temperature.

### Refinement

H atoms attached to toluene-ring C atoms were positioned geometrically and refined with a riding model.

### Crystal data

**Table:**

[Na(C_8_H_5_N_2_O_6_)]	*D*_x_ = 1.705 Mg m^−^^3^
*M**_r_* = 248.13	Mo *K*α radiation, λ = 0.71073 Å
Orthorhombic, *P**b**c**n*	Cell parameters from 25 reflections
*a* = 27.8428 (13) Å	θ = 6–15°
*b* = 10.452 (2) Å	µ = 0.18 mm^−^^1^
*c* = 6.642 (6) Å	*T* = 293 K
*V* = 1932.8 (17) Å^3^	Needle, yellow
*Z* = 8	0.42 × 0.14 × 0.08 mm
*F*(000) = 1008	

### Data collection

**Table:**

Kuma KM-4 four-circle diffractometer	1273 reflections with *I* > 2σ(*I*)
Radiation source: fine-focus sealed tube	*R*_int_ = 0.047
graphite	θ_max_ = 29.1°, θ_min_ = 1.5°
profile data from ω/2θ scans	*h* = 0→36
Absorption correction: analytical (*CrysAlis RED*; Oxford Diffraction, 2008)	*k* = −14→1
*T*_min_ = 0.975, *T*_max_ = 0.984	*l* = 0→9
2659 measured reflections	3 standard reflections every 200 reflections
2406 independent reflections	intensity decay: 0.01%

### Refinement

**Table:**

Refinement on *F*^2^	Primary atom site location: structure-invariant direct methods
Least-squares matrix: full	Secondary atom site location: difference Fourier map
*R*[*F*^2^ > 2σ(*F*^2^)] = 0.037	Hydrogen site location: inferred from neighbouring sites
*wR*(*F*^2^) = 0.132	H-atom parameters constrained
*S* = 1.00	*w* = 1/[σ^2^(*F*_o_^2^) + (0.0681*P*)^2^ + 0.6169*P*] where *P* = (*F*_o_^2^ + 2*F*_c_^2^)/3
2406 reflections	(Δ/σ)_max_ = 0.001
155 parameters	Δρ_max_ = 0.33 e Å^−^^3^
0 restraints	Δρ_min_ = −0.30 e Å^−^^3^

### Special details

**Table:**

Geometry. All e.s.d.'s (except the e.s.d. in the dihedral angle between two l.s. planes) are estimated using the full covariance matrix. The cell e.s.d.'s are taken into account individually in the estimation of e.s.d.'s in distances, angles and torsion angles; correlations between e.s.d.'s in cell parameters are only used when they are defined by crystal symmetry. An approximate (isotropic) treatment of cell e.s.d.'s is used for estimating e.s.d.'s involving l.s. planes.
Refinement. Refinement of *F*^2^ against ALL reflections. The weighted *R*-factor *wR* and goodness of fit *S* are based on *F*^2^, conventional *R*-factors *R* are based on *F*, with *F* set to zero for negative *F*^2^. The threshold expression of *F*^2^ > σ(*F*^2^) is used only for calculating *R*-factors(gt) *etc*. and is not relevant to the choice of reflections for refinement. *R*-factors based on *F*^2^ are statistically about twice as large as those based on *F*, and *R*- factors based on ALL data will be even larger.

### Fractional atomic coordinates and isotropic or equivalent isotropic displacement parameters (Å^2^)

**Table:**

	*x*	*y*	*z*	*U*_iso_*/*U*_eq_	
Na1	0.95606 (3)	−0.10192 (7)	0.92650 (12)	0.0318 (2)	
O2	0.96094 (6)	0.14635 (16)	1.0752 (2)	0.0389 (4)	
O11	0.75046 (8)	0.2906 (2)	0.8738 (4)	0.0747 (7)	
O1	0.93442 (7)	0.10334 (16)	0.7707 (3)	0.0482 (5)	
N1	0.77183 (7)	0.3679 (2)	0.9765 (4)	0.0427 (5)	
C1	0.90187 (7)	0.28318 (18)	0.9385 (3)	0.0246 (4)	
C7	0.93555 (7)	0.16846 (18)	0.9265 (3)	0.0268 (4)	
C5	0.89172 (7)	0.50978 (19)	0.9440 (3)	0.0273 (4)	
C3	0.82465 (7)	0.3756 (2)	0.9576 (3)	0.0294 (5)	
C2	0.85223 (7)	0.26469 (19)	0.9491 (3)	0.0283 (5)	
C4	0.84279 (7)	0.4982 (2)	0.9550 (3)	0.0287 (4)	
H4	0.8228	0.5695	0.9604	0.034*	
C6	0.92178 (8)	0.40496 (18)	0.9386 (3)	0.0273 (4)	
H6	0.9549	0.4157	0.9350	0.033*	
C8	0.83187 (9)	0.1325 (2)	0.9644 (5)	0.0479 (7)	
H8A	0.8039	0.1339	1.0489	0.072*	
H8B	0.8555	0.0762	1.0214	0.072*	
H8C	0.8232	0.1027	0.8326	0.072*	
O12	0.75280 (7)	0.4416 (2)	1.0929 (3)	0.0605 (6)	
N2	0.91229 (7)	0.63855 (17)	0.9404 (3)	0.0328 (4)	
O21	0.88557 (7)	0.72989 (15)	0.9215 (3)	0.0461 (5)	
O22	0.95584 (7)	0.64912 (16)	0.9575 (3)	0.0537 (5)	

### Atomic displacement parameters (Å^2^)

**Table:**

	*U*^11^	*U*^22^	*U*^33^	*U*^12^	*U*^13^	*U*^23^
Na1	0.0349 (5)	0.0263 (4)	0.0341 (5)	−0.0013 (3)	0.0000 (4)	0.0016 (3)
O2	0.0341 (9)	0.0433 (9)	0.0392 (9)	0.0091 (7)	−0.0041 (7)	0.0086 (7)
O11	0.0389 (9)	0.0753 (15)	0.1098 (19)	−0.0043 (11)	−0.0178 (13)	−0.0198 (15)
O1	0.0640 (12)	0.0410 (9)	0.0396 (10)	0.0191 (9)	0.0004 (9)	−0.0092 (8)
N1	0.0259 (9)	0.0409 (10)	0.0614 (14)	0.0058 (8)	−0.0002 (10)	0.0064 (11)
C1	0.0269 (9)	0.0237 (8)	0.0233 (10)	0.0025 (7)	−0.0026 (9)	0.0012 (8)
C7	0.0245 (9)	0.0250 (9)	0.0309 (11)	0.0025 (8)	0.0038 (9)	0.0057 (9)
C5	0.0373 (11)	0.0244 (9)	0.0204 (9)	−0.0007 (8)	−0.0007 (9)	0.0008 (8)
C3	0.0257 (10)	0.0349 (11)	0.0276 (10)	0.0022 (8)	−0.0011 (8)	−0.0009 (9)
C2	0.0275 (10)	0.0283 (10)	0.0292 (11)	−0.0004 (8)	−0.0018 (9)	0.0014 (9)
C4	0.0332 (9)	0.0294 (9)	0.0235 (10)	0.0085 (9)	0.0002 (9)	0.0016 (8)
C6	0.0291 (10)	0.0279 (9)	0.0247 (10)	−0.0015 (8)	−0.0004 (8)	0.0018 (9)
C8	0.0372 (12)	0.0314 (11)	0.0751 (19)	−0.0062 (10)	0.0010 (14)	0.0028 (12)
O12	0.0391 (9)	0.0542 (11)	0.0881 (15)	0.0131 (9)	0.0199 (11)	0.0039 (12)
N2	0.0467 (11)	0.0250 (8)	0.0266 (9)	−0.0013 (8)	0.0019 (9)	0.0017 (7)
O21	0.0611 (12)	0.0252 (7)	0.0518 (11)	0.0044 (7)	−0.0022 (10)	0.0003 (7)
O22	0.0440 (10)	0.0352 (9)	0.0819 (14)	−0.0116 (8)	0.0011 (10)	0.0017 (9)

### Geometric parameters (Å, °)

**Table:**

Na1—O1	2.4567 (19)	C1—C2	1.397 (3)
Na1—O2	2.780 (2)	C1—C7	1.524 (3)
Na1—O2^i^	2.3571 (17)	C5—C4	1.370 (3)
Na1—O1^ii^	2.364 (3)	C5—C6	1.379 (3)
Na1—O2^iii^	2.383 (3)	C5—N2	1.463 (3)
Na1—O22^iv^	2.6102 (19)	C3—C4	1.377 (3)
Na1—O21^iv^	2.635 (2)	C3—C2	1.392 (3)
Na1—Na1^i^	3.3881 (17)	C2—C8	1.497 (3)
Na1—Na1^v^	3.389 (2)	C4—H4	0.9300
Na1—Na1^iii^	3.946 (3)	C6—H6	0.9300
O2—C7	1.237 (3)	C8—H8A	0.9600
O2—Na1^i^	2.3571 (17)	C8—H8B	0.9600
O2—Na1^ii^	2.383 (3)	C8—H8C	0.9600
O11—N1	1.213 (3)	N2—O21	1.217 (2)
O1—C7	1.239 (3)	N2—O22	1.223 (2)
O1—Na1^iii^	2.364 (3)	N2—Na1^vi^	2.975 (2)
N1—O12	1.212 (3)	O21—Na1^vi^	2.635 (2)
N1—C3	1.478 (3)	O22—Na1^vi^	2.6102 (19)
C1—C6	1.388 (3)		
			
O2^i^—Na1—O1^ii^	104.67 (7)	C6—C1—C2	121.45 (18)
O2^i^—Na1—O2^iii^	84.31 (7)	C6—C1—C7	118.39 (18)
O1^ii^—Na1—O2^iii^	163.77 (7)	C2—C1—C7	120.16 (17)
O2^i^—Na1—O1	114.23 (7)	O2—C7—O1	125.38 (19)
O1^ii^—Na1—O1	110.50 (7)	O2—C7—C1	117.19 (18)
O2^iii^—Na1—O1	76.81 (7)	O1—C7—C1	117.40 (18)
O2^i^—Na1—O22^iv^	78.83 (6)	C4—C5—C6	122.34 (19)
O1^ii^—Na1—O22^iv^	85.23 (7)	C4—C5—N2	118.12 (18)
O2^iii^—Na1—O22^iv^	83.28 (7)	C6—C5—N2	119.53 (19)
O1—Na1—O22^iv^	154.57 (7)	C4—C3—C2	124.91 (19)
O2^i^—Na1—O21^iv^	126.78 (6)	C4—C3—N1	114.59 (18)
O1^ii^—Na1—O21^iv^	79.53 (6)	C2—C3—N1	120.47 (19)
O2^iii^—Na1—O21^iv^	84.27 (6)	C3—C2—C1	115.62 (18)
O1—Na1—O21^iv^	113.24 (7)	C3—C2—C8	123.86 (19)
O22^iv^—Na1—O21^iv^	48.26 (6)	C1—C2—C8	120.38 (18)
O2^i^—Na1—O2	97.91 (6)	C5—C4—C3	116.57 (19)
O1^ii^—Na1—O2	71.01 (6)	C5—C4—H4	121.7
O2^iii^—Na1—O2	121.81 (6)	C3—C4—H4	121.7
O1—Na1—O2	49.20 (6)	C5—C6—C1	119.07 (19)
O22^iv^—Na1—O2	154.51 (7)	C5—C6—H6	120.5
O21^iv^—Na1—O2	131.58 (6)	C1—C6—H6	120.5
C7—O2—Na1^i^	126.39 (14)	C2—C8—H8A	109.5
C7—O2—Na1^ii^	141.83 (14)	C2—C8—H8B	109.5
Na1^i^—O2—Na1^ii^	91.28 (6)	H8A—C8—H8B	109.5
C7—O2—Na1	82.10 (12)	C2—C8—H8C	109.5
Na1^i^—O2—Na1	82.09 (6)	H8A—C8—H8C	109.5
Na1^ii^—O2—Na1	99.40 (6)	H8B—C8—H8C	109.5
C7—O1—Na1^iii^	143.56 (16)	O21—N2—O22	123.02 (18)
C7—O1—Na1	97.00 (14)	O21—N2—C5	118.96 (19)
Na1^iii^—O1—Na1	109.83 (7)	O22—N2—C5	118.02 (17)
O12—N1—O11	124.5 (2)	N2—O21—Na1^vi^	93.83 (13)
O12—N1—C3	117.0 (2)	N2—O22—Na1^vi^	94.88 (13)
O11—N1—C3	118.5 (2)		

Symmetry codes: (i) −*x*+2, −*y*, −*z*+2; (ii) *x*, −*y*, *z*+1/2; (iii) *x*, −*y*, *z*−1/2; (iv) *x*, *y*−1, *z*; (v) −*x*+2, *y*, −*z*+3/2; (vi) *x*, *y*+1, *z*.
